# Supplementary material for: Theoretical Prediction on the New Types of Noble Gas Containing Anions OBONgO− and OCNNgO− (Ng=He, Ar, Kr and Xe)
Source: Molecules. 2020 Dec 10;25(24):5839. doi: 10.3390/molecules25245839 (PMC7763801; doi:10.3390/molecules25245839)
Supplement: Supplementary file 1 [file molecules-25-05839-s001.pdf]

Supplementary Materials for:

Theoretical Prediction on the New Types of Noble Gas Containing Anions $OBONgO^-$ and $OCNNgO^-$ (Ng = He, Ar, Kr and Xe)

Cheng-Cheng Tsai, Yu-Wei Lu and Wei-Ping Hu*
Department of Chemistry and Biochemistry, National Chung Cheng University, Chia-Yi 621, Taiwan
*Correspondence: chewph@ccu.edu.tw; Tel.: +886-5-272-0411 (ext. 66402)

3 Tables and 10 Figures

Submitted to *Molecules*, 2020

Table S1. The calculated three- and two-body dissociation energies, the two-body dissociation barriers, and the vertical singlet-triplet gaps of $NCONgO^{-}$. All energies are Born-Oppenheimer energies in kcal/mol.

$NCONgO^{-}$	NCO^{-} + Ng + O	Ng + $NC(O)_2^{-}$	Barrier	S–T gap
**Ng=He**				
MP2/apdz	13.2	–97.1	11.4	76.2
MP2/aptz	17.4	–97.0	14.7	88.0
CCSD(T)/aptz[a]	4.4[4.8]	–91.7[–92.1]	3.1	79.5[56.7]
CCSD(T)/apqz[a]	4.9	–91.8	9.2	80.2
**Ng=Ar**				
MP2/apdz	30.6	–79.7	19.6	38.1
MP2/aptz	39.3	–75.0	21.9	52.7
CCSD(T)/aptz[a]	23.9[24.9]	–72.2[–72.8]	17.2	40.0[30.7]
CCSD(T)/apqz[a]	24.2	–72.4	18.5	40.1
**Ng=Kr**				
MP2/apdz	49.6	–60.7	26.4	49.6
MP2/aptz	58.7	–55.7	28.0	61.4
CCSD(T)/aptz[a]	40.7	–55.5	23.5	49.0
CCSD(T)/apqz[a]	41.0	–55.6	23.7	49.0
**Ng=Xe**				
MP2/apdz	73.5	–36.8	33.2	58.1
MP2/aptz	84.5	–29.8	34.7	67.8
CCSD(T)/aptz[a]	63.2	–33.0	30.0	54.0
CCSD(T)/apqz[a]	63.8	–32.8	30.4	53.8

[a] Single-point calculation using MP2/apdz structures. For Ng = He and Ar, energies in brackets are obtained using CCSD(T)/aptz structures.

Table S2. The interconversion energetics from $NCONgO^{-}$ to $OCNNgO^{-}$. All energies are Born-Oppenheimer energies in kcal/mol.

$NCONgO^{-}$	$V^{\neq}$	$OCNNgO^{-}$
**Ng=Ar**		
MP2/apdz	1.9	–2.7
MP2/aptz	2.1	–3.0
CCSD(T)/aptz[a]	0.5	–1.2
CCSD(T)/apqz[a]	0.6	–2.3
**Ng=Kr**		
MP2/apdz	0.2	–3.5
MP2/aptz	3.4	–3.9
CCSD(T)/aptz[a]	1.3	–1.2
CCSD(T)/apqz[a]	1.5	–2.9
**Ng=Xe**		
MP2/apdz	5.0	–4.8
MP2/aptz	4.8	–5.2
CCSD(T)/aptz[a]	3.9	–2.1
CCSD(T)/apqz[a]	4.0	–3.9

[a]Single-point calculation using MP2/apdz structures

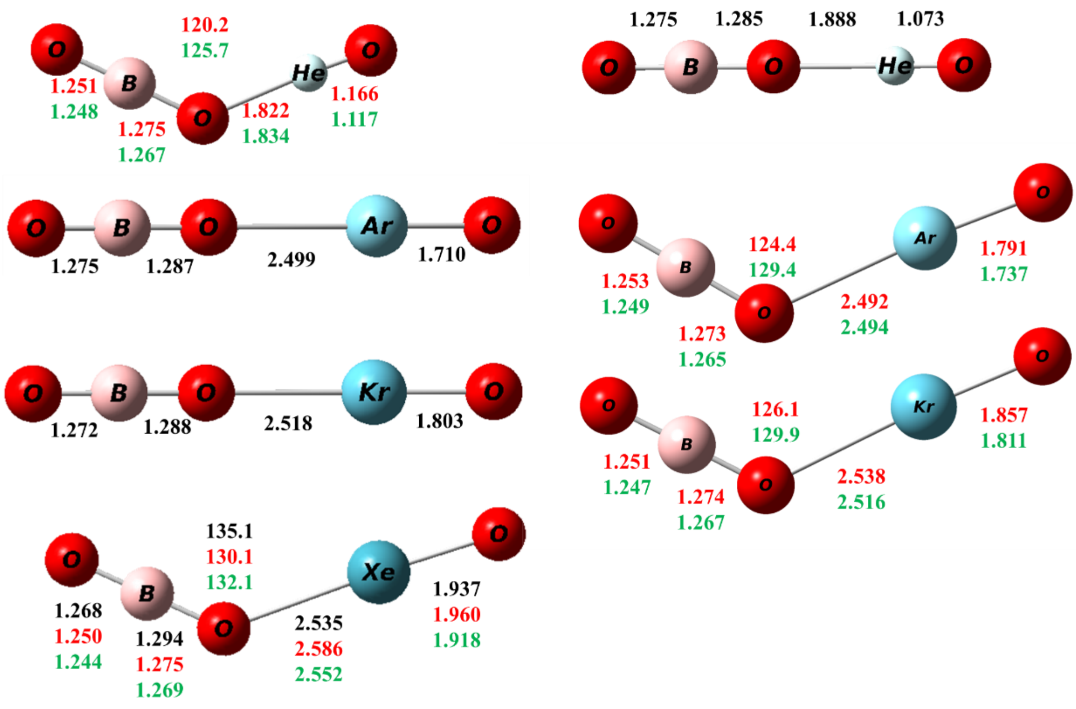

Figure S1. Calculated structures of $OBONgO^-$ (Ng = He, Ar, Kr and Xe). The bond distances are in angstroms and bond angles in degrees. The numbers in black, red, and green are values calculated by MP2/apdz, B3LYP/aptz, and MPW1B95/aptz methods, respectively.

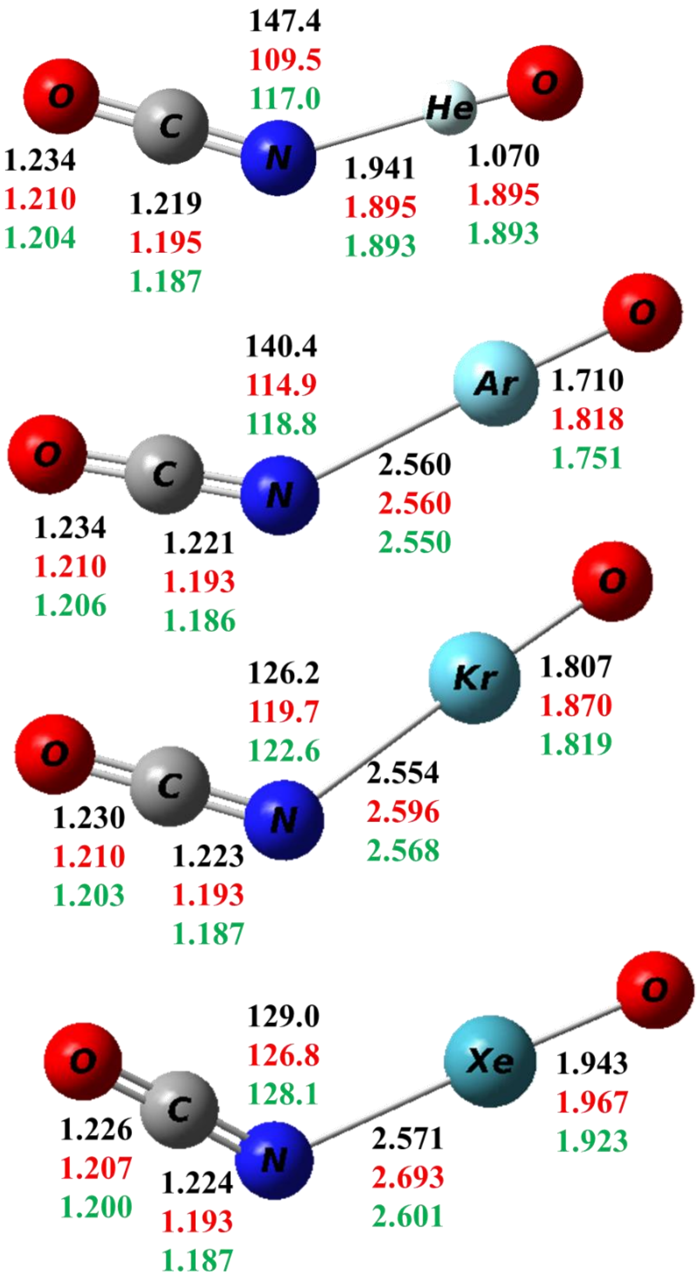

Figure S2. Calculated structures of $OCNNgO^-$ (Ng = He, Ar, Kr and Xe). The bond distances are in angstroms and bond angles in degrees. The numbers in black, red, green are values calculated by MP2/apdz, B3LYP/aptz, and MPW1B95/aptz methods, respectively.

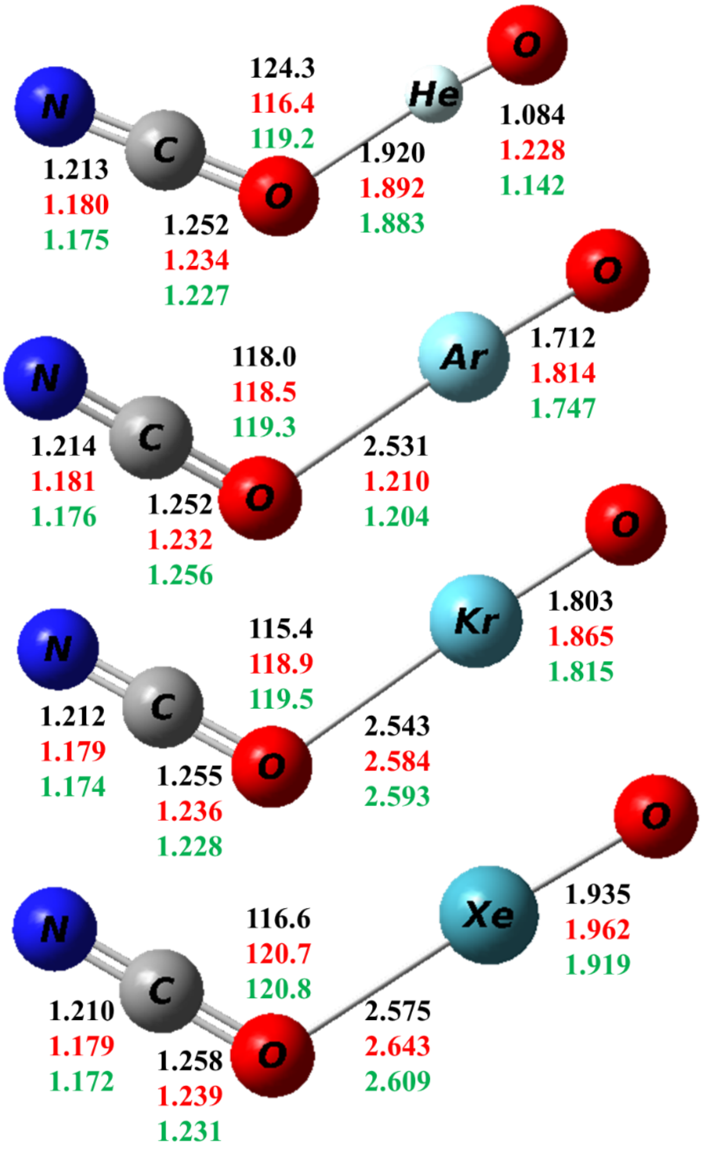

Figure S3. Calculated structures of $NCONgO^-$ (Ng = He, Ar, Kr and Xe). The bond distances are in angstroms and bond angles in degrees. The numbers in black, red, and green are values calculated by MP2/apdz, B3LYP/aptz, and MPW1B95/aptz methods, respectively.

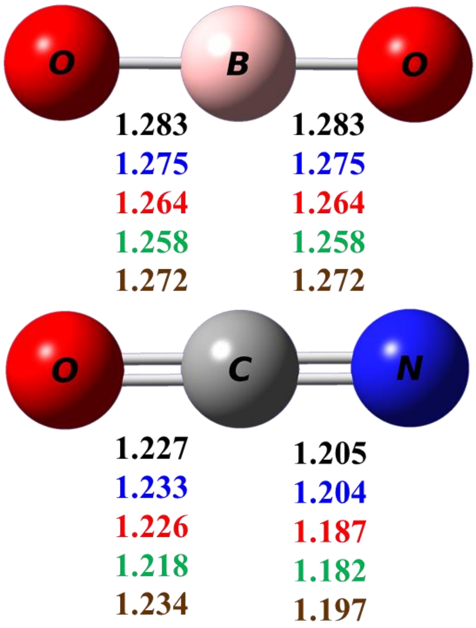

Figure S4. Calculated structures of OBO^- and OCN^-. The bond distances are in angstroms. The numbers in black, blue, red, green and brown are values calculated by MP2/apdz, MP2/aptz, B3LYP/aptz, MPW1B95/aptz, and CCSD(T)/aptz methods, respectively.

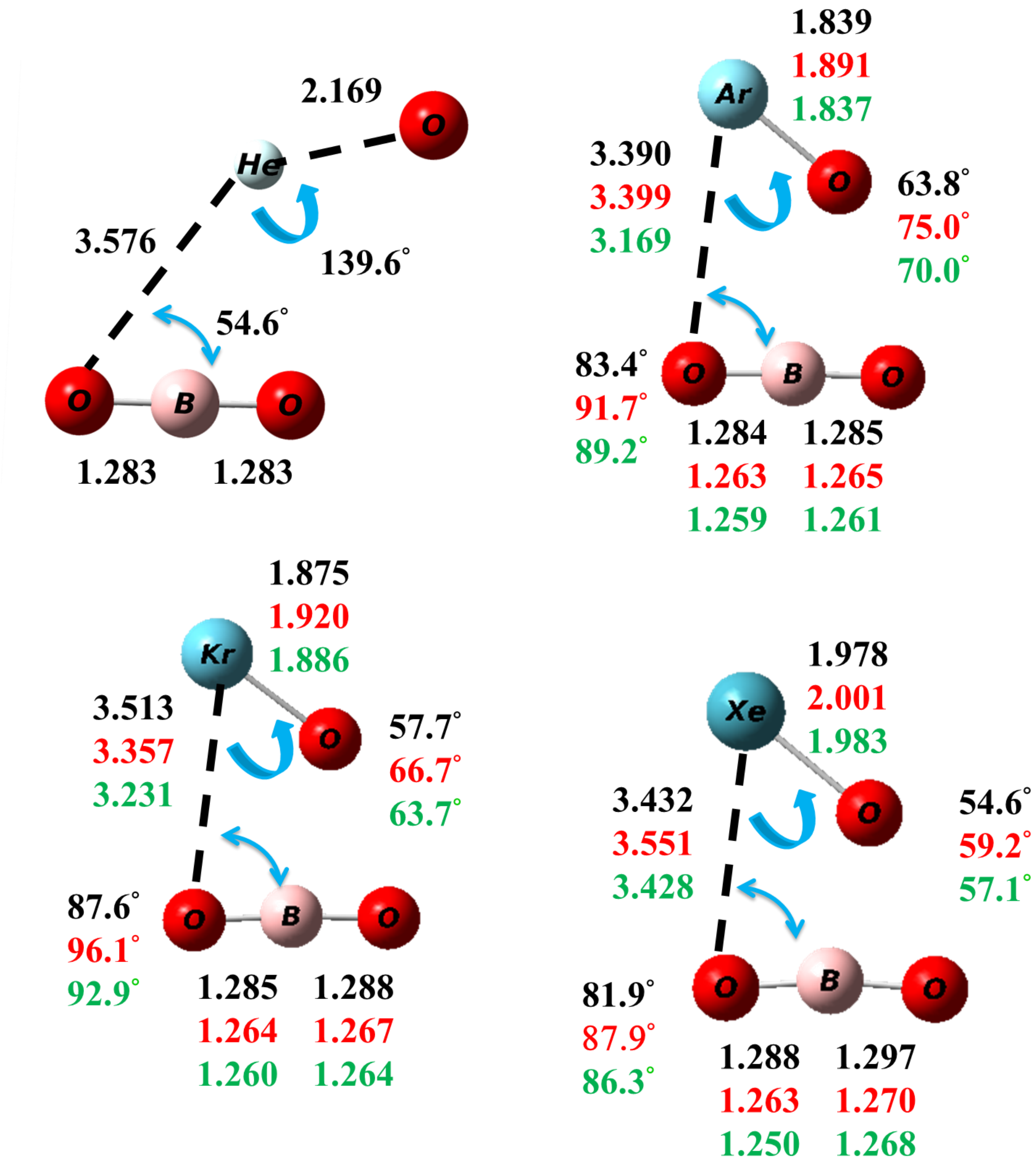

Figure S5. Calculated two-body dissociation transition state geometry of $OBONgO^{-}$ (Ng = He, Ar, Kr, and Xe). The bond lengths are in angstrom and bond angles in degrees. The black, blue, red and green values are calculated by MP2/apdz、MP2/aptz、B3LYP/aptz, and MPW1B95/aptz methods respectively.

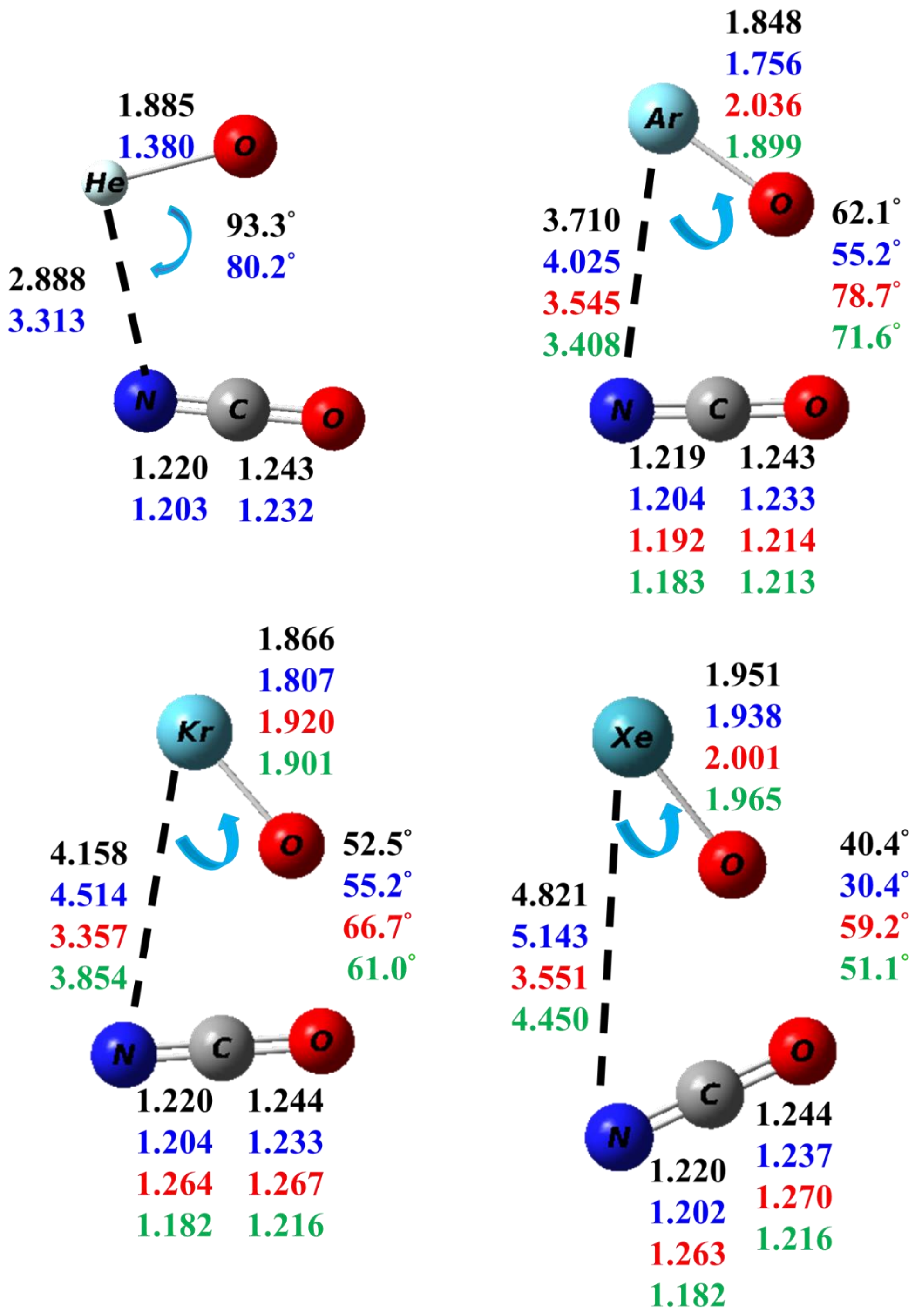

Figure S6. Calculated two-body dissociation transition state geometry of $OCNNgO^-$ (Ng = He, Ar, Kr, and Xe). The bond lengths are in angstrom and bond angles in degrees. The black, blue, red and green values are calculated by MP2/apdz, MP2/aptz, B3LYP/aptz, and MPW1B95/aptz methods respectively.

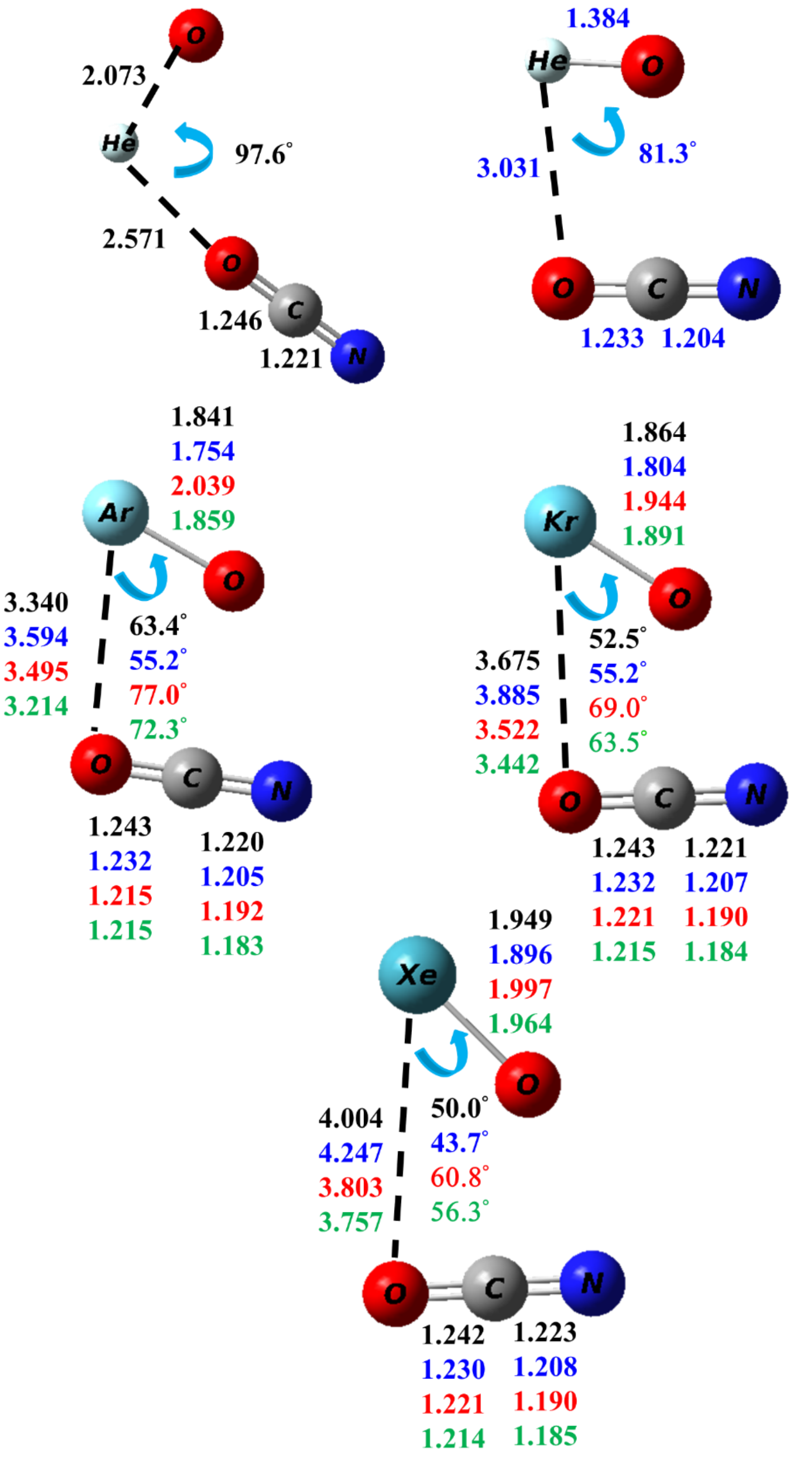

Figure S7. Calculated two-body dissociation transition state geometry of $NCONgO^-$ (Ng = He, Ar, Kr, and Xe). The bond lengths are in angstrom and bond angles in degrees. The black, blue, red and green values are calculated by MP2/apdz, MP2/aptz, B3LYP/aptz, and MPW1B95/aptz methods respectively.

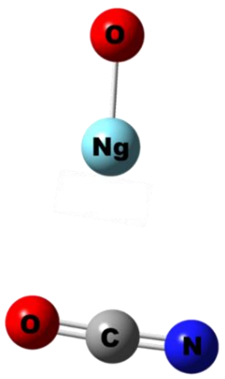

Figure S8. Transition state structure for the interconversion from OCNNgO⁻ to OCNNgO⁻.

Table S3. Calculated transition state geometries as shown in Figure S8 (bond length in Å, angle in degrees) for the interconversion reaction from NCONgO⁻ to OCNNgO⁻.

	MP2/apdz	MP2/aptz	B3LYP/ aptz	MPW1B95/ aptz
Ng=*Ar*				
R(O-Ar)	1.721	1.672	1.778	1.738
R(O-C)	1.246	1.235	1.228	1.221
R(C-N)	1.218	1.203	1.186	1.18
R(Ar-C)	2.913	2.867	2.962	2.929
A(O-C-N)	177.4	177.2	178	177.9
A(Ar-C-N)	97.9	97.8	99.4	99.1
Ng=*Kr*				
R(O-Ar)	1.807	1.768	1.851	1.814
R(O-C)	1.246	1.235	1.228	1.221
R(C-N)	1.218	1.203	1.185	1.18
R(Kr-C)	2.957	2.911	3.041	2.989
A(O-C-N)	177	176.9	177.8	177.6
A(Kr-C-N)	97.0	97.0	98.7	98.4
Ng=*Xe*				
R(O-Xe)	1.91	1.892	1.955	1.921
R(O-C)	1.246	1.235	1.229	1.221
R(C-N)	1.219	1.203	1.185	1.18
R(Xe-C)	3.009	2.984	3.13	3.063
A(O-C-N)	176.5	176.4	177.4	177.1
A(Xe-C-N)	96.2	96.2	97.9	97.5

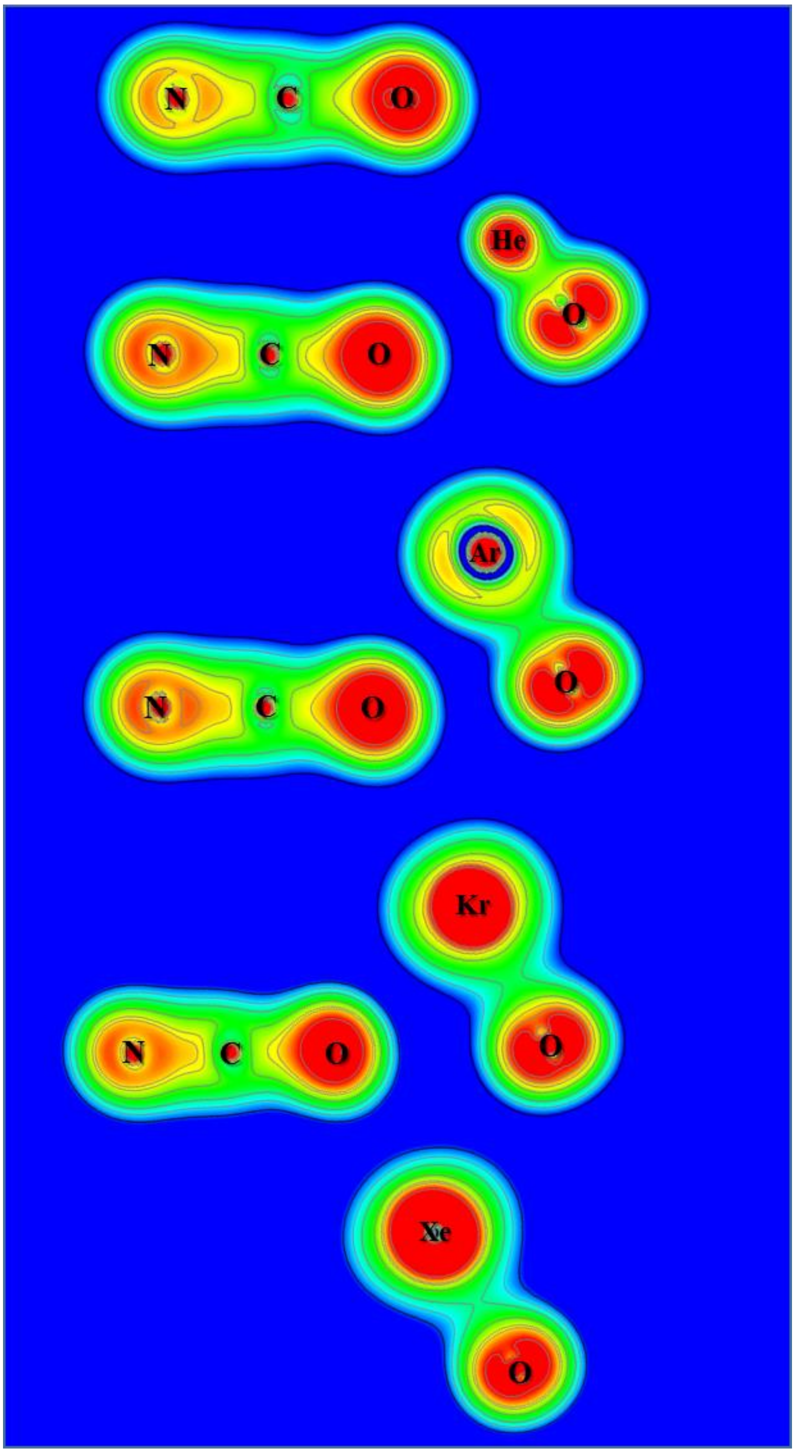

Figure S9. Contour plots of the calculated electron density of NCONgO⁻

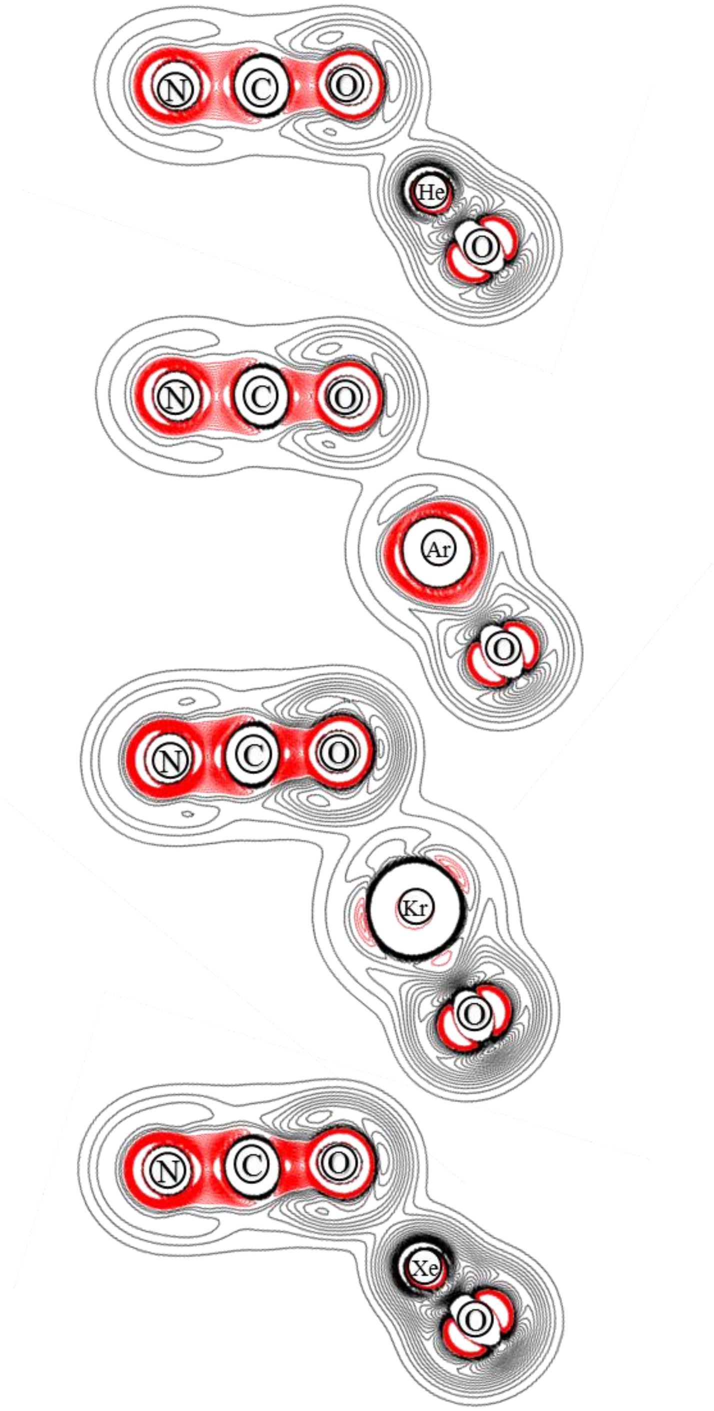

Figure S10. Contour plots of the calculated Laplace concentration of $NCONgO^{-}$. The red contour lines are in regions of charge concentration and the black contour lines are in regions of charge depletion.
